# A Nanopore Structured High Performance Toluene Gas Sensor Made by Nanoimprinting Method

**DOI:** 10.3390/s100100765

**Published:** 2010-01-21

**Authors:** Kwang-Su Kim, Woon-Hyuk Baek, Jung-Min Kim, Tae-Sik Yoon, Hyun Ho Lee, Chi Jung Kang, Yong-Sang Kim

**Affiliations:** 1 Department of Nano Science and Engineering, Myongji University, Gyeonggi 449-728, Korea; E-Mails: 9515king@mju.ac.kr (K.-S.K.); whbaek@mju.ac.kr (W.-H.B.); jmkim122@mju.ac.kr (J.-M.K.); tsyoon@mju.ac.kr (T.-S.Y.); cjkang@mju.ac.kr (C.J.K.); 2 Department of Chemical Engineering, Myongji University, Gyeonggi 449-728, Korea; E-Mail: hyunho@mju.ac.kr; 3 Department of Electrical Engineering, Myongji University, Gyeonggi 449-728, Korea

**Keywords:** toluene, gas sensor, MIS structure, nanoimprinting method, titania, nanopore

## Abstract

Toluene gas was successfully measured at room temperature using a device microfabricated by a nanoimprinting method. A highly uniform nanoporous thin film was produced with a dense array of titania (TiO_2_) pores with a diameter of 70∼80 nm using this method. This thin film had a Pd/TiO_2_ nanoporous/SiO_2_/Si MIS layered structure with Pd-TiO_2_ as the catalytic sensing layer. The nanoimprinting method was useful in expanding the TiO_2_ surface area by about 30%, as confirmed using AFM and SEM imaging. The measured toluene concentrations ranged from 50 ppm to 200 ppm. The toluene was easily detected by changing the Pd/TiO_2_ interface work function, resulting in a change in the *I–V* characteristics.

## 1. Introduction

The presence of toxic and pollutant gases, such as toluene (C_7_H_8_), carbon monoxide, carbon dioxide, NO_x_ *etc.*, at home and in the workplace, in addition to outdoors, poses occupational and health hazards, and thus gas sensing devices need to be installed in such places. These devices should be reagentless, cheap, and able to quantify the level of gases in a rapid manner, preferably at room temperature with low power consumption. Gaseous toluene is highly toxic and originates from paint and varnish thinners commonly used in construction [1]. Existing methods of gas detection such as gas chromatography [2] are not only insensitive to parts per billion (ppb) levels of toluene but also cannot be installed into households. In the past, various attempts have been made to develop a toluene gas sensor [3], but these sensors exhibited poor sensing performance. For example, they required a high gas concentrations for detection and high operational temperatures (50∼500 °C).

The surface morphology of the sensing layer closely influences the sensitivity of a gas sensor [4]. Various groups have attempted to expand the surface area of the gas sensing layer using various photolithographic techniques and advanced materials such as SWCNTs (single walled carbon nanotubes) or MWCNTs (multi walled carbon nanotubes) [5–8]. Although these reports garnered considerable interest because of the faster response times of the resulting gas sensors, these materials present poor recovery periods at room temperature while sensing toluene, CO, CO_2_, NO_x_, *etc*. Therefore, a sensing material with better recovery rates at room temperature must be found, without sacrificing the device sensitivity and response time.

For these reasons, this study attempted to develop a toluene gas sensor that was operational at room temperature. The configuration of this sensor adopted a catalytic-metal-insulator-semiconductor (MIS) structure. A catalytic Pd-TiO_2_ thin film was chosen as the gas sensing layer because of its known ability to detect volatile organic compounds, including toluene [9], at a low operational temperature [4–8]. Also, Pd-TiO_2_ is less expensive and less toxic than other sensing layers such as CdS, GaN, SnO_2_, In_2_O_3_, *etc.* commonly used in the fabrication of gas sensors [10,11,15,17–19]. Additionally, Pd has higher catalytic action than typical catalysts such as gold (Au) and platinum (Pt) [10,12]. The key mechanism of the sensor is the reactivity of the Pd-TiO_2_ film with toluene gas. At the Pd surface, the hydrocarbon molecules are dissociated into smaller molecules and diffused through the Pd to the Pd/TiO_2_ interface. Consequently, this phenomenon creates a dipole layer that changes the work function of the Pd/TiO_2_ interface. The formation of the dipole layer is analyzed through the changes in the *R-V* characteristics in presence of different concentrations of toluene. In the present case, toluene gas decreases the resistance level, and the changes are proportional to the toluene concentration [9]. In addition to this sensing principle, a novel nanoimprinting method was attempted to improve the sensitivity of the sensor by increasing the surface area of the sensing layer.

## 2. Experimental Section

### 2.1. Fabrication

**Figure 1.**
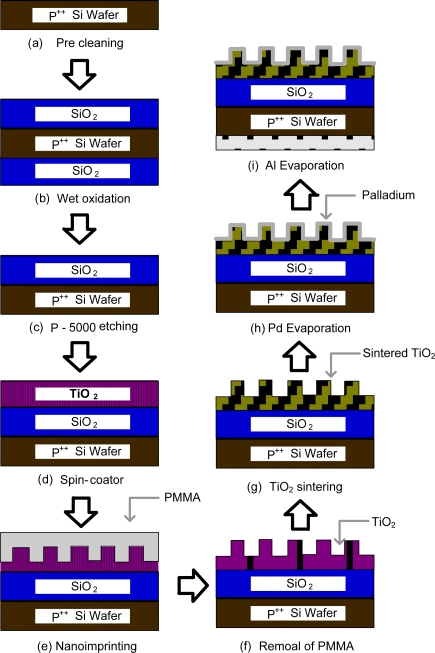
Schematic diagram for the gas sensor fabrication steps: (a) Silicon wafer precleaning, (b) wet oxidation in a furnace, (c) back-side of the wafer was etched using the P-5000 solution, (d) TiO_2_ layer spin-coating, (e) nano-imprinting with a premade PMMA patterned mold, (f) removing the PMMA mold, (g) TiO_2_ layer sintering over 500 °C, (h) Palladium layer depositing by evaporation, (i) evaporating Al on the backside of wafer to form the ohmic contact.

The catalytic MIS structure of the Pd-TiO_2_ thin film gas sensor was fabricated on low resistivity p-type or n-type heavily doped Si as Pd/TiO_2_ nanoporous/SiO_2_/Si layers with a surface area of 1.3 × 1.3 cm^2^. Figure 1 shows the schematic diagram of the device fabrication steps. First the Si was cleaned with sulfuric-peroxide mixture (SPM) to remove the native oxide layer, and then a 100 nm thick SiO_2_ insulator layer was grown on the Si using wet oxidation. The backside of the Si was etched using reactive ion etching to form the ohmic contact. The surface morphology of the TiO_2_ and Pd affects the device performance. Therefore, a nanoimprinting process [13] was used to pattern the anodic aluminum oxide (AAO) to expand the surface area. Figure 1 also shows the fabrication procedure of the well-ordered nanopored titania structures. For this fabrication, an Al plate (1 × 3 cm^2^) was first degassed? in acetone and electropolished in a 1:5 mixed solution of perchloric (60%) acid and ethanol (85%) under a constant voltage condition (15 V) at 3 °C for 2 min to create a mirrored surface. Al was anodized at a constant voltage of 50 V in a 0.3 M oxalic acid solution at 15 °C for 4 h. Thereafter, the anodic oxide layer was removed using a mixture of phosphoric acid (50 mL, 85%), chromic acid (18 g, Aldrich) and deionized water (950 mL) at 60 °C for 2 h. The second anodization step was performed under the same conditions for 40∼60 sec. The pores were widened by dipping the nanoporous alumina template in a 0.5 M phosphoric acid solution for 1 hour.

Polymethyl methacrylate (PMMA, Aldrich) with a molecular mass of 350 kg/mol was dissolved in chlorobenzene and poured onto the nanoporous alumina template to fabricate a nanopoled polymer. The PMMA infiltrated into the nanopores during heating at 150 °C. The PMMA nanopoles formed after the alumina template was removed through wet etching in a 1.4 wt% FeCl_3_/5 M HCl solution, and the remaining Al and alumina was removed with a 10 wt% NaOH solution. The titania sol-gel solution was prepared by mixing titanium (IV) ethoxide (1.5 g), HCl (3 g, 38%) and 2-propanol (10 g). The titania sol-gel solution was spin-coated onto a SiO_2_/Si, and then the substrate was embossed with the PMMS nanopoles before the solution was dried. The embossed titania film was sintered in a dry oven at 100 °C for 1 hour. The nanoporous titania film was obtained by removing the PMMA nanopoles with acetonitrile. Subsequently, a Pd electrode with a thickness of 60 nm and an area of 1 cm^2^ was thermally evaporated using a shadow mask on the pored TiO_2_ surface. A high current was applied for 600 sec for the deposition using a thermal evaporator because Pd has a high melting point, *i.e.*, above 1,500 °C. Finally, a metal Al contact, with a thickness of 0.1 μm, was evaporated on the opposite side of the device as an ohmic contact.

### 2.2. Spectroscopic and microscopic characterizations

The surface morphology of the titania film was analyzed with an atomic force microscope (AFM, Parksystems, XE-100) using a Pt-Ir coated SiN_3_ tip under the non-contact mode. The composition of the titania films was determined using semiquantitative energy-dispersive X-ray (EDX, Hitachi, S-3500N) analysis. The crystalline phase of the titania films prepared under various conditions was determined using an X-ray diffraction instrument. For this analysis, the titania layer was prepared over the ITO surface [13] at different sintering temperatures. The X-ray diffraction (XRD) diffractograms of these layers were compared with titania powder to analyze the possible presence of the peak related to the anatase phase. A field emission scanning electron microscope (FE-SEM) was also used to examine the cross section of the gas sensor device. For this analysis, the device was first coated with Pt and inserted in the FE-SEM chamber under a vacuum of 10^−5^ torr.

### 2.3. Gas detection system

A gas chamber was designed with a provisional gas flow through a Mass Flow Controller (MFC) with a flow rate range of 1 sccm to 100 sccm. The working volume of the gas chamber was approximately 2 L. There were two gas flow lines within the chamber. One line was used to supply the gas and the other line was used to collect gas samples in a Tedlar® bag in order to confirm the concentration of the gases with a gas chromatography (GC) unit. Before the target gases were injected, nitrogen was injected into the chamber to remove any other gases present and to create atmospheric conditions (STP). The electronic interface of the detection unit had a Keithley^®^ 236 voltage source and a current measurement meter that could supply (–3 V) static and (–3 to 0 V) sweep voltages. In this study, the static potential mode was used to determine the sensing time, and the sweep voltage was used to obtain the *I-V* curve.

## 3. Results and Discussion

### 3.1. Analysis of TiO_2_ film

**Figure 2.**
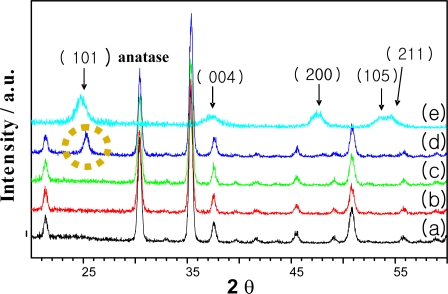
XRD data: (a) ITO, (b) Sintered at 200 °C, (c) 350 °C, (d) 500 °C, (e) TiO_2_ powder.

Titania is a well studied transition metal oxide. In the last few years, interest in both the application and the fundamental research of this material has increased because of its remarkable optical and electronic properties and its good catalytic activity, which is similar to those of Au and Pt [9,13,14]. The thickness and roughness of the TiO_2_ layer in a gas sensor significantly influence the sensitivity of device [9,14]. Therefore, studying the surface morphology of TiO_2_ layer was important. The XRD spectra of the TiO_2_ film (Figure 2) revealed the effect of the sintering temperature on the film formation. The anatase peak in this figure was seen at about 25 degrees. This phase has been shown to be linked with gas sensor sensitivity in previous reports [9,16]. Also, anatase (101) was not present under a sintering temperature of 500 °C. Therefore, processing of the device as maintained over 500 °C for 1 hour to form the TiO_2_ anatase phase seen in Figure 2.

### 3.2. Analysis of Pd/TiO_2_ nanoporous layer

**Figure 3.**
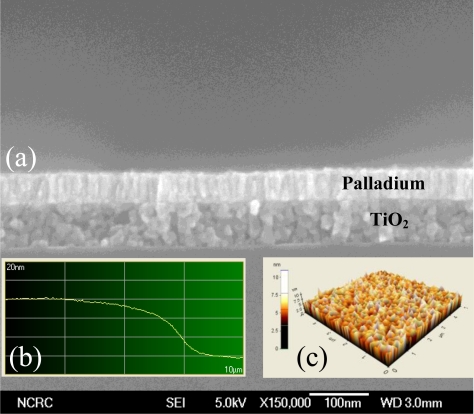
Surface & device thickness analysis: (a) FE-SEM image, (b) AFM, (c) morphology from AFM.

**Figure 4.**
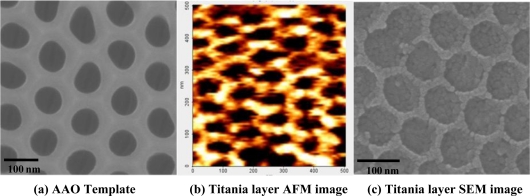
Nanopore image: (a) AAO template using FE-SEM, (b) imprinted Titania layer AFM image, (c) FE-SEM image.

The roughness and the thickness of Pd layer that was deposited using a thermal evaporator affected the gas sensing performance in a similar manner to TiO_2_. Therefore, the cross sectional images of non-nanoimprinted device were examined using FE-SEM (Figure 3), and revealed a palladium thickness of about 60 nm. In the latter part of the study, the nanoimprinting method was used to expand the surface area of the sensing layer. Therefore, the device fabricated with the nanopored TiO_2_ layer had a higher surface roughness than the non-nanopored process (flat surface). The palladium surface in these devices was monitored using the AFM lithography process. Figure 4 shows the topography of the Pd surface monitored using AFM and FE-SEM. The AAO template (Figure 4a) was prepared to make the PMMA mold. The imprinted TiO_2_ nanopore size was about 70∼90 nm, as seen in Figure 4b. The SEM image of the imprinted TiO_2_ layer (Figure 4c) illustrated the surface morphology. From these data, the surface area of the imprinted TiO_2_ layer was larger than the non-imprinted layer.

### 3.3. Detection of nitrogen gas (with non-imprinted Pd layer)

First, the fabricated devices with the non-imprinted Pd layer were placed in the gas chamber and tested for detection of N_2_. The detection was performed under a low vacuum and under a standard air condition (80%:20% N_2_:O_2_ respectively). The standard diagnostic test of the sensor operation was a voltage sweep from the negative to the positive direction while measuring the current. During this diagnosis, the devices showed the typical Schottky diode *I-V* characteristics, as previously reported [3,9]. N_2_ and O_2_ gases decrease the sensors’ output current level because these gases react with palladium and TiO_2_ on the gas sensor device [9]. The experiments were repeated on the device with nitrogen gas that was diluted with standard air. Various concentrations of nitrogen ranging between 50 ppm to 200 ppm were injected into the detection chamber to determine *R-V* characteristics. The gas sensor’s resistance increased proportionately with the N_2_ concentration. The nitrogen gas sensor operated typically in the forward bias region at a bias of 1.5∼1.8 V, with a current change from 47 mA to 67 mA at an N_2_ flow rate of 100 sccm. The difference in the sensor output with nitrogen gas compared to standard air was 20 mA with a forward bias of 1.7 V. The sensor response time was only 2 minutes.

### 3.4. Detection of toluene gas with non-imprinted Pd layer

**Figure 5.**
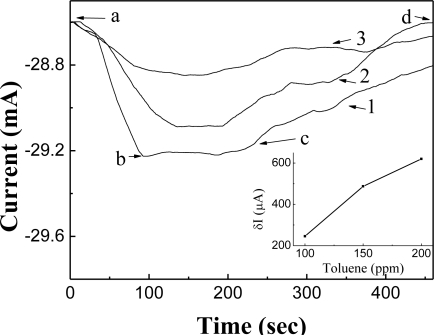
Realtime characteristics (reponse and recovery data) for a toluene concentration of: (1) 200 ppm, (2) 150 ppm, and (3) 100 ppm. (a) Injection of toluene gas into the gas chamber, (b) sturation, (c) benting toluene gas through the gas line(out), and (d) recovery.

The gas sensor with the non-imprinted Pd layer was subsequently tested to detect toluene in N_2_ using the same reaction chamber. The adsorption of toluene gas at the Pd-TiO_2_ interface of the sensor resulted in a change of the gas sensor’s *I-V* characteristics, and the relationship between the sensor output and toluene concentration was linear (Figure 5). However, the sensor output in terms of current level for the toluene gas injection was smaller compared to nitrogen, but the gas sensing characteristics were excellent in the former case. This gas sensor operated even at room temperature, and the detection time was only a few minutes. Additionally, the sensor was reused with a full recovery to the baseline level (Figure 5, stretch-d) by drawing the air out of the vessel after the reaction reached steady state (Figure 5, stretch-b to c) by pumping fresh air into the chamber. At high toluene concentrations, the response time was shorter for longer recovery times, and *vice versa*. The higher recovery period for the device could be reduced by applying an opposite voltage [9,10].

### 3.5. Detection of toluene gas with nanoimprinted Pd layer

**Figure 6.**
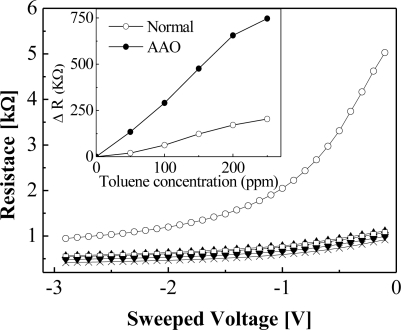
Comparison of the imprinted and non-imprinted device operating characteristic.

**Figure 7.**
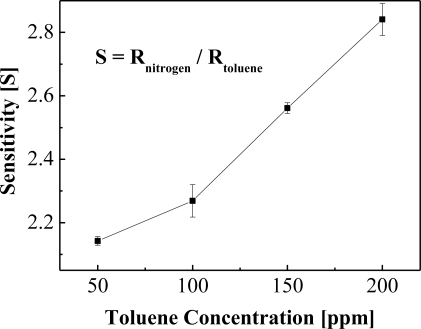
Characteristics of sensitivity with the toluene gas concentrations.

In addition to the operability of the sensor at room temperature, this study also attempted to improve the sensitivity of the gas sensor device towards toluene. Therefore, the surface appearance was changed using a nanoimprinting method to effectively enhance the surface roughness (to about 30%), and the gas sensing characteristics improved due to an increased interface reaction between the palladium surface and the charged toluene ions. The nanoimprinted device was more sensitive towards toluene gas than the device fabricated using a non-imprinted (normal) method as evident in Figure 6, thereby validating the relationship between the surface roughness and the device sensitivity. On the other hand, the sensitivity of the AAO device, in Equation 1 where R is the change in the resistance from the baseline, was not affected by increasing the toluene concentration (Figure 7) [7–9]:

(1) $$S=R_{\text{nitrogen}}/R_{\text{toluene}}$$

In other words, the device had a higher sensitivity at a low toluene concentration while using the AAO nanoimprinting method.

## 4. Conclusions

Fabrication using a nanoimprinting process of a new toluene gas sensor that was operable at room temperature was proposed. The sensor had a silicon diode structure comprised of Pd/TiO_2_ nanoporous/SiO_2_/Si. According to measured current levels at low applied voltage, the device could detect a concentration of toluene. Nowadays, semiconductor-based devices are typically operated from 25 °C to 300 °C. Our device could operate in room temperature and detect with high sensitivity.
